# Relationship between Prognostic Stage in Breast Cancer and Fluorine-18 Fluorodeoxyglucose Positron Emission Tomography/Computed Tomography

**DOI:** 10.3390/jcm10143173

**Published:** 2021-07-19

**Authors:** Mio Mori, Tomoyuki Fujioka, Kazunori Kubota, Leona Katsuta, Yuka Yashima, Kyoko Nomura, Emi Yamaga, Junichi Tsuchiya, Tokuko Hosoya, Goshi Oda, Tsuyoshi Nakagawa, Iichiroh Onishi, Ukihide Tateishi

**Affiliations:** 1 Department of Diagnostic Radiology, Tokyo Medical and Dental University, 1-5-45 Yushima, Bunkyo-ku, Tokyo 113-8510, Japan; m_mori_116@yahoo.co.jp (M.M.); fjokmrad@tmd.ac.jp (T.F.); leonah@jcom.home.ne.jp (L.K.); 11.ruby.89@gmail.com (Y.Y.); nomura.kyoko@kameda.jp (K.N.); ymgdrnm@tmd.ac.jp (E.Y.); tuwu11@gmail.com (J.T.); ttisdrnm@tmd.ac.jp (U.T.); 2 Department of Radiology, Dokkyo Medical University Saitama Medical Center, 2-1-50 Minamikoshigaya, Koshigaya, Saitama 343-8555, Japan; 3 Department of Surgery, Breast Surgery, Tokyo Medical and Dental University, 1-5-45 Yushima, Bunkyo-ku, Tokyo 113-8510, Japan; tocco_nkgw@yahoo.co.jp (T.H.); oda.srg2@tmd.ac.jp (G.O.); nakagawa.srg2@tmd.ac.jp (T.N.); 4 Department of Comprehensive Pathology, Tokyo Medical and Dental University, 1-5-45 Yushima, Bunkyo-ku, Tokyo 113-8510, Japan; iichpth2@tmd.ac.jp

**Keywords:** breast cancer, positron emission tomography, standard uptake value, prognostic stage, American Joint Committee on Cancer

## Abstract

This retrospective study examined the relationship between the standardized uptake value max (SUVmax) of fluorine-18 fluorodeoxyglucose positron emission tomography/computed tomography (^18^F-FDG PET/CT) and the prognostic stage of breast cancer. We examined 358 breast cancers in 334 patients who underwent ^18^F-FDG PET/CT for initial staging between January 2016 and December 2019. We extracted data including SUVmax of ^18^F-FDG PET and pathological biomarkers, including estrogen receptor (ER), progesterone receptor (PR), human epidermal growth factor receptor 2 (HER2), and nuclear grade. Anatomical and prognostic stages were determined per the American Joint Committee on Cancer (eighth edition). We examined whether there were statistical differences in SUVmax between each prognostic stage. The mean SUVmax values for clinical prognostic stages were as follow: stage 0, 2.2 ± 1.4; stage IA, 2.6 ± 2.1; stage IB, 4.2 ± 3.5; stage IIA, 5.2 ± 2.8; stage IIB, 7.7 ± 6.7; and stage III + IV, 7.0 ± 4.5. The SUVmax values for pathological prognostic stages were as follows: stage 0, 2.2 ± 1.4; stage IA, 2.8 ± 2.2; stage IB, 5.4 ± 3.6; stage IIA, 6.3 ± 3.1; stage IIB, 9.2 ± 7.5, and stage III + IV, 6.2 ± 5.2. There were significant differences in mean SUVmax between clinical prognostic stage 0 and ≥II (*p* < 0.001) and I and ≥II (*p* < 0.001). There were also significant differences in mean SUVmax between pathological prognostic stage 0 and ≥II (*p* < 0.001) and I and ≥II (*p* < 0.001). In conclusion, mean SUVmax increased with all stages up to prognostic stage IIB, and there were significant differences between several stages. The SUVmax of ^18^F-FDG PET/CT may contribute to prognostic stage stratification, particularly in early cases of breast cancers.

## 1. Introduction

Breast cancer staging is used to ascertain the extent of the disease, help implement a treatment plan, and predict prognosis [1]. Since 1977, the American Joint Committee on Cancer (AJCC) has published a staging system for breast cancer based on anatomic findings, including tumor size (T), nodal status (N), and metastases (M) [2]. The eighth edition of the AJCC staging system introduced a new prognostic staging system that incorporates biomarkers such as estrogen receptor (ER), progesterone receptor (PR), and human epidermal growth factor receptor 2 (HER2) status, and tumor grade [2]. Since the publication of this new prognostic staging system, several studies have reported its correlation with prognosis in clinical settings [1,3,4]. The prognostic stage should be used as a primary staging system in countries where these biomarker tests are routinely performed for patient care, and cancer registries in the United States must use the prognostic stage group tables for reporting cases [2].

^18^F-fluorodeoxyglucose positron emission tomography/computed tomography (^18^F-FDG PET/CT) has the advantage of being able to evaluate both the morphology and glucose metabolism of the lesions [5]. It is useful in breast cancers to identify distant metastases or secondary cancers and is also used to predict prognosis and therapeutic effects [6,7,8,9,10]. Previous studies have shown that the maximum standardized uptake value (SUVmax), a common semiquantitative value of ^18^F-FDG PET/CT, correlates with ER, PR, HER2, and tumor grade [11,12,13]. However, the relationship between the AJCC prognostic stage, which is a complex concept including T, N, M, and biomarkers, and ^18^F-FDG PET/CT remains unclear.

This retrospective study examined whether the SUVmax of ^18^F-FDG PET/CT contributes to the stratification of the prognostic stage. This is the first study to investigate the relationship between prognostic stage and ^18^F-FDG PET/CT findings.

## 2. Materials and Methods

### 2.1. Study Design and Patients

This retrospective study enrolled consecutive patients who underwent ^18^F-FDG PET/CT for initial staging for breast cancer between January 2016 and December 2019. Among 699 ^18^F-FDG PET/CT analyses performed for breast cancer patients during the study period, 361 ^18^F-FDG PET/CT were excluded because they were performed to evaluate recurrent cancers and assess therapeutic efficacy. Altogether, 338 ^18^F-FDG PET/CT analyses performed for initial staging were included. Four cancers of four patients were excluded, as the biomarkers required to determine both clinical and pathological prognostic stages were not pathologically diagnosed. Among the remaining 334 patients, 20 had two tumors and 2 had three tumors. A final total of 334 patients with 358 breast cancers were enrolled. Three patients had a history of contralateral breast cancer surgery. The remaining 331 patients had no history of breast-related disease.

Among the 314 patients who underwent surgery as an initial treatment, the mean interval between ^18^F-FDG PET/CT and surgery was 28.1 ± 23.6 days (range, 2–210 days), and among the 329 breast cancers biopsied prior to ^18^F-FDG PET/CT, the mean interval was 22.1 ± 11.3 days (range, 0–112 days).

### 2.2. ^18^F-FDG PET/CT Protocol

^18^F-FDG (3.7 MBq/kg; 0.1 mCi/kg) was administered intravenously in all patients after at least a 4 h fasting period. Whole-body images were then routinely obtained using a PET/CT system (Aquiduo or Celesteion, Canon Medical Systems, Tochigi, Japan). CT was also performed using the following parameters: pitch, 0.938; gantry rotation time, 0.5 s; table time, 30 mm/s; auto-exposure control (SD 20), 120 KVp; and slice thickness, 2.0 mm. Contrast materials were not used for CT examinations. Whole-body emission PET was performed after approximately 60 min of ^18^F-FDG administration using the following parameters: emission time per bed, 2 min; bed position, 9–10; slice thickness, 4.08 mm; and matrix, 144 × 144.

### 2.3. ^18^F-FDG PET/CT Analysis

SUVmax data for the breast cancers were obtained from diagnostic reports provided by nuclear medicine experts, with more than eight years of experience, who performed the initial staging of breast cancer. A circular region of interest (ROI) was set on the highest uptake of the primary breast cancer to calculate SUVmax using Vox-base version 2.8 (J-MAC SYSTEM, Inc., Hokkaido, Japan). The ROI was set avoiding nipples, which are areas with high physiological accumulation. When the accumulation of lesions was low and distinguishing the lesion from the background mammary gland was difficult, the position of ROI was determined by referring to contrast-enhanced breast magnetic resonance imaging. All images were measurable and had few motion artifacts.

### 2.4. Clinicopathological Evaluation

Age, gender, and pathological information of the patients were obtained from medical records.

Surgical specimens of breast tissue were cut by pathologists into 5–10-mm contiguous sections, and thinner slices were added as required. All surgical and biopsy cases were diagnosed by a pathologist who recorded the following histological features: tumor size of invasive component; nuclear grade (1, 2, or 3); and receptor status (ER, PR, and HER2). AJCC recommends using histological grade as the tumor grade; however, nuclear grade was used in this study. A cutoff of 1% was used for ER and PR assays according to the American Society of Clinical Oncology/College of American Pathologists guidelines [14]. HER2 status was defined as positive if 3+ on immunohistochemistry or fluorescence in situ hybridization demonstrated gene amplification [15].

### 2.5. Anatomical and Prognostic Stages

The anatomical stage was based solely on the anatomical extent of the cancer, as defined by the T, N, and M categories [2]. Staging of the 314 cancers was performed using available surgical specimens, and the remaining 44 were staged using whole-body ^18^F-FDG PET/CT, contrast-enhanced breast magnetic resonance imaging, and breast ultrasonography, as either only biopsy specimens were available or neoadjuvant therapy was given. A pathological prognostic stage was used for patients who had undergone surgical resection as the initial treatment for their cancer before receiving systemic or radiation therapy and was based on tumor grade and ER, PR, and HER2 status from surgery and resected tissue as well as T, N, and M stage [2]. A clinical prognostic stage was applied to cancers with available biopsy specimens. Among the 358 cancers, a pathological prognostic stage was determined in 313 and a clinical prognostic stage was determined in 323. The relationship between the stages is shown in Table 1 and Table 2 [2,16].

### 2.6. Statistical Analysis

We examined whether the prognostic stage could be stratified by SUVmax alone, irrespective of TNM stage and biomarker status. The clinical and pathological prognostic stages were grouped as follows: stage 0, I, II, and III + IV. First, the Kolmogorov–Smirnov test was used to examine if the variables were normally distributed. The Kruskal–Wallis test, which is a nonparametric test, was performed to examine whether the null hypothesis was rejected by some groups, assuming normality. In the subsequent Dunn–Bonferroni test, we examined whether there was a significant difference in mean SUVmax between the stages. All statistical analyses were performed using IBM SPSS Statistics version 24 (International Business Machines Corporation, Armonk, NY, USA). *p*-Values < 0.05 were considered to indicate statistical significance. Data are presented as mean SUVmax ± standard deviation.

## 3. Results

### 3.1. Patients

A final total of 358 breast cancers in 334 patients were enrolled. Among the 334 patients, 20 had two cancers and 2 had three tumors. All patients were female, and the mean age was 58.9 ± 12.9 years (range, 26–87 years). Surgery revealed invasive ductal carcinomas of no special type (*n* = 225), ductal carcinoma in situ (*n* = 31), invasive lobular carcinoma (*n* = 22), mucinous carcinoma (*n* = 13), invasive micropapillary carcinoma (*n* = 7), apocrine carcinoma (*n* = 6), mixed invasive ductal and lobular carcinoma (*n* = 3), lobular carcinoma in situ (*n* = 2), Paget’s disease (*n* = 2), squamous cell carcinoma (*n* = 1), microinvasive carcinoma (*n* = 1), and papillary carcinoma (*n* = 1). The remaining 44 lesions were diagnosed with invasive ductal carcinomas of no special type (*n* = 35), invasive lobular carcinoma (*n* = 4), mucinous carcinoma (*n* = 1), apocrine carcinoma (*n* = 1), ductal carcinoma (*n* = 1; presence or absence of invasion was unknown), invasive carcinoma (*n* = 1; unknown tissue subtype), and invasive ductal carcinoma with metaplastic carcinoma component by biopsy (*n* = 1). The clinical prognostic stage was determined in 323 cancers, and the pathological prognostic stage was determined in 313 cancers. The biomarker status of the cancers is shown in Table 3. HER2 status or nuclear grade was unknown in some cases, but cases in which the missing status did not change the prognostic stage were included.

### 3.2. Anatomical and Prognostic Stages

Table 4 and Table 5 show the number of cancers assigned to each stage. According to the AJCC criteria, 33 cancers (10.2%) were upstaged, 206 cancers (63.8%) remained unchanged, and 84 cancers (26.0%) were downstaged from anatomical stage to clinical prognostic stage. In addition, 17 cancers (5.4%) were upstaged, 201 cancers (64.2%) remained unchanged, and 95 cancers (30.4%) were downstaged from anatomical stage to pathological prognostic stage [2].

### 3.3. SUVmax at Each Prognostic Stage

**Figure 1:**
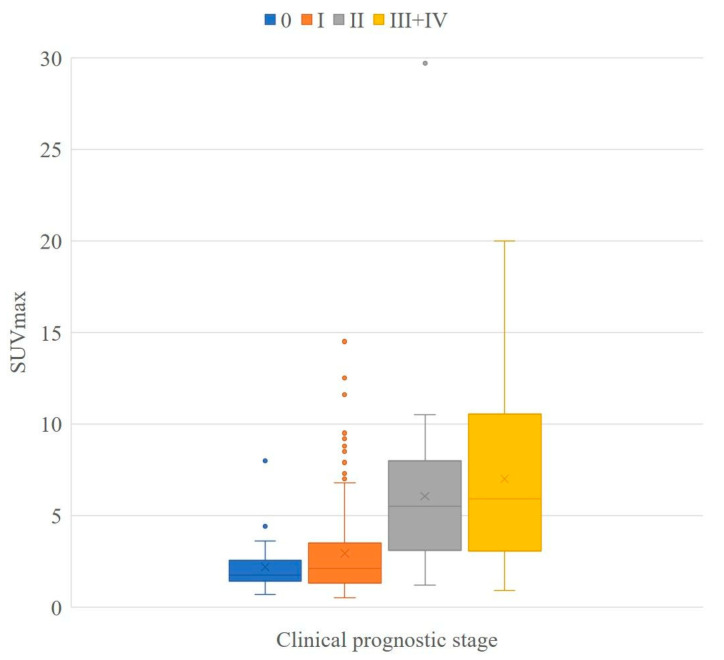
Boxplot of clinical prognostic stage. Boxplots showing the distribution of SUV max for each clinical prognostic stage. Stage III and IV are summarized at the right end of the graph owing to the small sample size at each stage. The sample size for each stage was 36 for stage 0, 198 for stage I, 43 for stage II, and 46 for stage III + IV.

**Figure 2:**
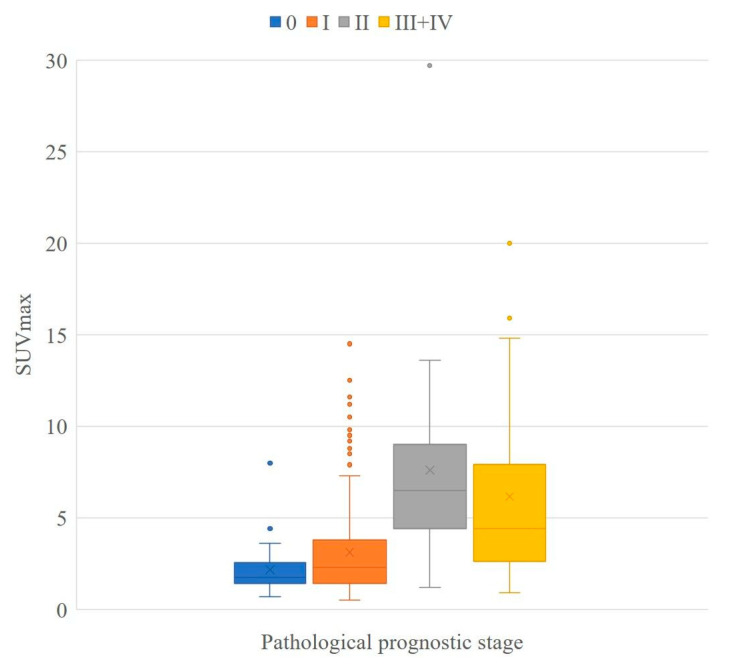
Boxplot of pathological prognostic stage. Boxplots showing the distribution of SUV max for each pathological prognostic stage. Stage III and IV are summarized at the right end of the graph owing to the small sample size at each stage. The sample size for each stage was 36 for stage 0, 231 for stage I, 26 for stage II, and 20 for stage III + IV.

The mean SUVmax values for the clinical prognostic stage were found to be 2.2 ± 1.4 (stage 0), 2.6 ± 2.1 (stage IA), 4.2 ± 3.5 (stage IB), 5.2 ± 2.8 (stage IIA), 7.7 ± 6.7 (stage IIB), 7.9 ± 6.0 (stage IIIA), 5.8 ± 4.2 (stage IIIB), 9.4 ± 6.0 (stage IIIC), 7.3 ± 2.6 (stage IV), and 7.0 ± 4.5 (stage III + IV) (Table 6). The mean SUVmax values for the pathological prognostic stage were found to be 2.2 ± 1.4 (stage 0), 2.8 ± 2.2 (stage IA), 5.4 ± 3.6 (stage IB), 6.3 ± 3.1 (stage IIA), 9.2 ± 7.5 (stage IIB), 5.4 ± 3.9 (stage IIIA), 3.0 ± 0.5 (stage IIIB), 11.7 ± 7.8 (stage IIIC), 5.3 (stage IV), and 6.2 ± 5.2 (stage III + IV) (Table 4). Boxplots of the SUVmax for each prognostic stage are shown in Figure 1 and Figure 2.

The Kolmogorov–Smirnov test did not show normal distribution in stage 0–II of the clinical prognostic stage (*p* < 0.001–0.009) and 0–III + IV (*p* < 0.001–0.022) of the pathological prognostic stage. The Kruskal–Wallis test showed that there was a significant difference in both the clinical and pathological prognostic stages (*p* < 0.001). There were significant differences in the mean SUVmax between the clinical prognostic stage 0 and ≥II (*p* < 0.001) and I and ≥II (*p* < 0.001). There were also significant differences in the mean SUVmax between the pathological prognostic stage 0 and ≥II (*p* < 0.001) and I and ≥II (*p* < 0.001) (Table 7).

**Figure 3:**
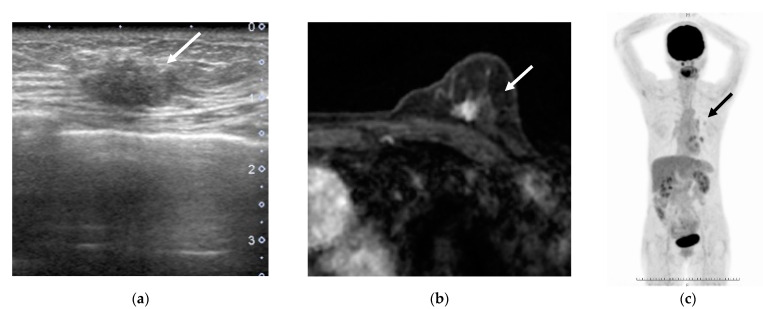
A woman in her 60s was found to have a left invasive ductal carcinoma of no special type by surgery (arrows). (**a**) Breast ultrasonography (B mode), (**b**) contrast-enhanced breast magnetic resonance image (axial image of early phase of contrast-enhanced), and (**c**) whole-body positron emission tomography. Pathological analysis revealed T2N0M0, nuclear grade 1, positive estrogen receptor, positive progesterone receptor, and negative human epidermal growth factor receptor 2 (not shown). She was found to have an SUVmax of 1.4, anatomical stage IIA, clinical prognostic stage IB, and pathological prognostic stage IA.

**Figure 4:**
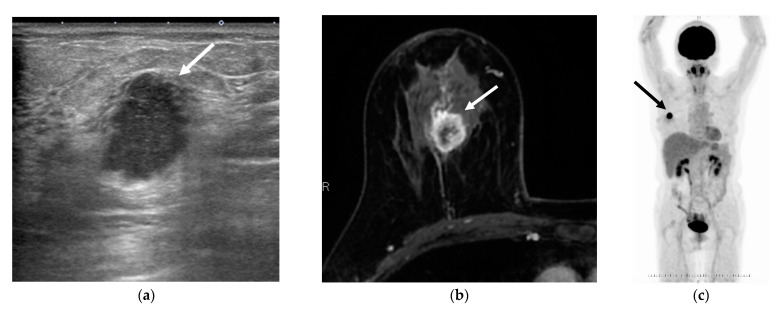
A woman in her 60s was found to have a right invasive ductal carcinoma of no special type by surgery (arrows). (**a**) Breast ultrasonography (B mode), (**b**) contrast-enhanced breast magnetic resonance image (axial image of early phase of contrast-enhanced), and (**c**) whole-body positron emission tomography. Pathological analysis revealed T1cN0M0, nuclear grade 3, negative estrogen receptor, negative progesterone receptor, and negative human epidermal growth factor receptor 2 (not shown). She was found to have an SUVmax of 12.5, anatomical stage IA, clinical prognostic stage IB, and pathological prognostic stage IB.

Some cases with low tumor SUVmax were downstaged from the anatomical to the prognostic stage (Figure 3), and some cases with high tumor SUVmax were upstaged from the anatomical stage to the prognostic stage (Figure 4).

## 4. Discussion

The present study revealed that the mean SUVmax increased with all stages up to prognostic stage IIB, and there were significant differences in mean SUVmax between clinical prognostic stages 0 and ≥II, and I and ≥II. There were also significant differences in mean SUVmax between pathological prognostic stage 0 and ≥II, and I and ≥II. The SUVmax of ^18^F-FDG PET/CT may contribute to the stratification of the prognostic stage, particularly in early breast cancers. To our knowledge, this is the first study to investigate the relationship between prognostic stage and ^18^F-FDG PET/CT findings.

It has long been reported that biomarkers, including ER, PR, HER2, and tumor grade, correlate with prognosis [17,18,19,20]; the AJCC recently published a prognostic staging system that incorporates this concept [2]. The incorporation of biomarkers into the prognostic staging system results in stage migration for certain patients [21]. For example, triple-negative tumors are generally “upstaged” in the prognostic stage, whereas HER2 expression is a “downstaging” factor [2,21]. Abdel-Rahman reported that all pairwise hazard ratio comparisons between different stages were significant among 209,304 patients according to prognostic stages [4]. Weiss et al. reported that the prognostic stage provided more accurate stratification with respect to disease-specific survival than the anatomic stage among 3327 patients with stage I–IIIC and 54,727 patients with stage I–IV breast cancer [1]. Wang et al. reported that the prognostic stage was an independent prognostic factor among 10,053 locally advanced breast cancers according to multivariate analysis [3]. These findings highlight the importance of the prognostic stage rather than the anatomical stage for prognosis prediction and treatment strategy. However, the prognostic stage is based on pathological findings, and preoperative diagnostic imaging alone is not sufficient.

^18^F-FDG PET/CT is a non-invasive approach that shows morphological findings as well as metabolic activities associated with malignancy [5,6]. Several studies have reported the usefulness of ^18^F-FDG PET/CT for predicting prognosis in breast cancer. Kadoya et al. reported that univariate and multivariate analyses identified high SUVmax as an independent prognostic factor among 344 patients with clinical stage I–III breast cancer, using a SUVmax cutoff value of 3.0 [6]. A meta-analysis showed that breast cancer patients with high SUVmax were at high risk of adverse events or death, with high metabolic tumor volume predicting a high risk of death and high total lesion glycolysis predicting a high risk of adverse events [9]. Previous studies suggest that high FDG uptake is correlated with bigger tumor size, negative ER expression, negative PR expression, positive HER2 expression, and high histological grade [11,12,13]. HER2 status is conflicting, as while HER2 positivity is a good prognostic factor, it is associated with a high SUVmax. There is still no consensus on how each biomarker affects SUVmax when combined.

In the present study, there was no change from anatomical stage to clinical prognostic stage in 63.8% of cases and no change from anatomical stage to pathological prognostic stage in 64.2% of cases (Table 3 and Table 4). Anatomical and prognostic stages can be considered to be very different staging systems. The prognostic stage is an ideal staging system for the prediction of prognosis, whereas pathological diagnosis is required, and the correspondence table is complicated. There is often a difference between the biopsy and surgical pathology results. In the present study, two cases were upstaged in the clinical prognostic stage but downstaged in the pathological prognostic stage, and one case showed the opposite findings. We focused on ^18^F-FDG PET/CT as a tool to stratify prognostic staging. The SUVmax increased as the stage increased up to prognostic stage IIB, and there was a significant difference between some stages (Table 6 and Table 7, Figure 1 and Figure 2). HER2 status, which shows conflicting results for high SUVmax and good prognostic potential, did not appear to be a problem in the present study. It is possible that ER, PR, and nuclear grade mitigated the effects of HER2 status. Furthermore, since the present study included consecutive cases during the study period, there were few HER2-positive cancers. Verifying whether prognosis worsens with an increase in the SUVmax of HER2-positive breast cancers is essential. The mean SUVmax was found to be higher in stage I than in stage 0; however, no significant difference was observed between stage 0 and I in the clinical and pathological prognostic stages (Table 6 and Table 7). This does not appear to be important from a prognostic point of view because the five-year survival rate for the prognostic stage I is very good (above 97.5%) [1]. In addition, it was difficult to demonstrate a significant difference in the higher stages of II vs. III + IV (Table 7). This may be because lymph node status is more important than the glucose metabolic activity of primary breast cancer in the higher stages; therefore, evaluation of SUVmax in the primary lesion may be insufficient, and total primary and metastatic lesion glycolysis, which is calculated by multiplying the metabolic tumor volume of a lesion with its corresponding mean SUV, may be a better indicator of prognosis [22]. Another reason for these findings may be that only a small number of cases of higher stages were included in this study. Additional studies with increased numbers are required.

One limitation of the present study is that it was retrospective in nature. Second, it had a relatively small sample size. The number of types of breast cancers included in this study may have affected the results, but statistical analysis based on types was not possible owing to the small sample size. Third, while the AJCC recommends histological grade as the tumor grade, this study used nuclear grade. Fourth, we did not examine whether uptake of ^18^F-FDG correlates with actual prognosis. However, this correlation has been extensively discussed previously and we examined its association with the prognostic stage in the present study. Finally, there is an opinion that ^18^F-FDG PET/CT is not required for early-stage breast cancer. However, it has been reported that up to 44% of patients diagnosed with ductal carcinoma in situ by biopsy develop invasive cancer [23], and it may not be possible to assert early-stage breast cancer by biopsy alone. Furthermore, as this study suggested that the prognostic stage is associated with SUVmax, it may be recommended that ^18^F-FDG PET/CT be performed to estimate the malignancy of breast cancer.

## 5. Conclusions

SUVmax of ^18^F-FDG PET/CT may allow stratification of the prognostic stage, particularly in early breast cancers. This non-invasive and simple method may be clinically useful and allow tumor properties to be evaluated to predict the prognosis of breast cancer patients.

## Figures and Tables

**Table 1:** Correspondence table showing anatomical and clinical prognostic stages.

		Clinical Prognostic Stage					Anatomical Stage
TNM	Grade	Triple Positive HR+, HER2+	Luminal-Like HR+, HER2-	HER2-Like HR±, HER2+	HR±, HER2-	Triple Negative HR-, HER2-	
TisN0	G1–3	0	0	0	0	0	0
T1N0 T0–1N1mi	G1/G2 G3	IA	IA	IA	IA	IB	IA(/IB)
		IA	IA	IA	IA/IB(ER-)	IB	
T1N1 T2N0	G1/G2 G3	IB	IB	IIA	IIA	IIA/IIB(G2)	IIA
		IB	IIA	IIA	IIB	IIB	
T2N1 T3N0	G1/G2 G3	IB	IIA	IIA/IIB(HR-)	IIB	IIB/IIIB(G2)	IIB
		IB	IIB	IIB	IIIA	IIIB	
T0–3N2 T3N1	G1/G2 G3	IIA	IIA	IIIA	IIIA	IIIB	IIIA
		IIB	IIIA	IIIA	IIIB	IIIC	
T4N0–2 Any N3	G1/G2 G3	IIIA	IIIB	IIIB	IIIB	IIIC	IIIB, IIIC
		IIIB	IIIB	IIIB	IIIC	IIIC	
Any M1	G1–3	IV	IV	IV	IV	IV	IV

G, nuclear grade; HER2+, HER2 receptor-positive; HER2-, HER 2 receptor-negative; HR+, estrogen receptor-positive/progesterone receptor-positive; HR-, estrogen receptor-negative/progesterone receptor-negative; HR±, estrogen receptor-positive/progesterone receptor-negative and estrogen receptor-negative/progesterone receptor-positive; Tis, carcinoma in situ. Gray, stage 0; Pale yellow, stage IA; Yellow, Stage IB; Pale green, stage IIA; Green, stage IIB; Pale pink, stage IIIA; Pink, stage IIIB; Vivid pink, stage IIIC; Blue, stage IV.

**Table 2:** Correspondence table showing anatomical and pathological prognostic stages.

		Clinical Prognostic Stage					Anatomical Stage
TNM	Grade	Triple Positive HR+, HER2+	Luminal-Like HR+, HER2-	HER2-Like HR±, HER2+	HR±, HER2-	Triple Negative HR-, HER2-	
TisN0	G1–3	0	0	0	0	0	0
T1N0 T0-1N1mi	G1/G2 G3	IA	IA	IA	IA	IA/IB(G2)	IA(/IB)
		IA	IA	IA	IA	IB	
T1N1 T2N0	G1/G2 G3	IA	IA	IB/IIA(HR-)	IB/IIA(G2)	IIA	IIA
		IA	IA	IIA	IIA	IIA	
T2N1 T3N0	G1/G2 G3	IA/IB(G2)	IA/IB(G2)	IIB	IIB	IIB	IIB
		IB	IIA	IIB	IIB	IIIA	
T0–3N2 T3N1	G1/G2 G3	IB	IB	IIIA	IIIA	IIIA/IIIB(G2)	IIIA
		IIA	IIB	IIIA	IIIA	IIIC	
T4N0–2 Any N3	G1/G2 G3	IIIA	IIIA	IIIB	IIIB	IIIB/IIIC(G2)	IIIB, IIIC
		IIIB	IIIB	IIIB	IIIC	IIIC	
Any M1	G1–3	IV	IV	IV	IV	IV	IV

G, nuclear grade; HER2+, HER2 receptor-positive; HER2-, HER 2 receptor-negative; HR+, estrogen receptor-positive/progesterone receptor-positive; HR-, estrogen receptor-negative/progesterone receptor-negative; HR±, estrogen receptor-positive/progesterone receptor-negative and estrogen receptor-negative/progesterone receptor-positive; Tis, carcinoma in situ. Gray, stage 0; Pale yellow, stage IA; Yellow, Stage IB; Pale green, stage IIA; Green, stage IIB; Pale pink, stage IIIA; Pink, stage IIIB; Vivid pink, stage IIIC; Blue, stage IV.

**Table 3:** Clinicopathological findings.

		Anatomical Stage (*n* = 358)	Clinical Prognostic Stage (*n* = 323)	Pathological Prognostic Stage (*n* = 313)
Age (years)		58.9 ± 12.9	58.9 ± 12.9	59.0 ± 12.9
T	Tis	37 (10.3%)	36 (11.1%)	36 (11.5%)
	1	196 (54.7%)	175 (54.2%)	186 (59.4%)
	2	100 (27.9%)	88 (27.2%)	82 (26.2%)
	3	13 (3.6%)	12 (3.7%)	9 (2.9%)
	4	12 (3.4%)	12 (3.7%)	0 (0%)
N	0	248 (69.3%)	225 (69.7%)	232 (74.1%)
	1	72 (20.1%)	64 (19.8%)	57 (18.2%)
	2	17 (4.7%)	15 (4.6%)	16 (5.1%)
	3	21 (5.9%)	19 (5.9%)	8 (2.6%)
M	0	348 (97.2%)	313 (96.9%)	312 (99.7%)
	1	10 (2.8%)	10 (3.1%)	1 (0.3%)
ER	+	283 (79.1%)	255 (78.9%)	256 (81.8%)
	-	75 (20.9%)	68 (21.1%)	57 (18.2%)
PR	+	261 (72.9%)	236 (73.1%)	237 (75.7%)
	-	97 (27.1%)	87 (26.9%)	76 (24.3%)
HER2	+	52 (14.5%)	47 (14.6%)	41 (13.1%)
	-	283 (79.1%)	256 (79.3%)	252 (80.5%)
	Unknown	23 (6.4%)	20 (6.2%)	20 (6.4%)
Nuclear grade	1	185 (51.7%)	168 (52.0%)	161 (51.4%)
	2	68 (19.0%)	66 (20.4%)	58 (18.5%)
	3	97 (27.1%)	82 (25.4%)	84 (26.8%)
	Unknown	8 (2.2%)	7 (2.2%)	10 (3.2%)

The table shows the clinicopathological findings for each group. Age is presented as mean ± SD; other measurements are presented as number (%). ER, estrogen receptor; HER2, human epidermal growth factor receptor; M, metastasis status; N, nodal status; PR, progesterone receptor; T, tumor status; Tis, carcinoma in situ.

**Table 4:** Migration from anatomical to clinical prognostic stage.

		Clinical Prognostic Stage									
		0	IA	IB	IIA	IIB	IIIA	IIIB	IIIC	IV	Total
**Anatomical** **stage**	**0**	36 *									36
	**IA**		135 *	10							145
	**IB**		4								4
	**IIA**		2	44	7 *	8		1			62
	**IIB**		1	2	16	6 *		4			29
	**IIIA**				6		3 *	5	2		16
	**IIIB**							5 *	3		8
	**IIIC**							9	4 *		13
	**IV**									10 *	10
	**Total**	36	142	56	29	14	3	24	9	10	323

* Number of cancers that did not migrate.

**Table 5:** Migration from anatomical to pathological prognostic stage.

		Pathological Prognostic Stage									
		0	IA	IB	IIA	IIB	IIIA	IIIB	IIIC	IV	Total
**Anatomical** **stage**	**0**	36 *									36
	**IA**		144 *	8							152
	**IB**		4								4
	**IIA**		48	7	11 *	1					67
	**IIB**		7	8	3	6 *	4				28
	**IIIA**			5		5	3 *		4		17
	**IIIB**										
	**IIIC**						4	4			8
	**IV**									1 *	1
	**Total**	36	203	28	14	12	11	4	4	1	313

* Number of cancers that did not migrate.

**Table 6:** SUVmax of each stage.

	Clinical Prognostic Stage (*n* = 323)	Pathological Prognostic Stage (*n* = 313)
**0**	2.2 ± 1.4 (36)	2.2 ± 1.4 (36)
**IA**	2.6 ± 2.1 (142)	2.8 ± 2.2 (203)
**IB**	4.2 ± 3.5 (36)	5.4 ±3.6 (28)
**IIA**	5.2 ± 2.8 (29)	6.3 ± 3.1 (14)
**IIB**	7.7 ± 6.7 (14)	9.2 ± 7.5 (12)
**IIIA**	7.9 ± 6.0 (3)	5.4 ± 3.9 (11)
**IIIB**	5.8 ± 4.2 (24)	3.0 ± 0.5 (4)
**IIIC**	9.4 ± 6.0 (9)	11.7 ± 7.8 (4)
**IV**	7.3 ± 2.6 (10)	5.3 (1)
III + IV *	7.0 ± 4.5 (46) *	6.2 ± 5.2 (20) *

Mean SUVmax ± standard deviation (number of cancers). * Stages III and IV were present in low numbers and were combined.

**Table 7:** Statistical analysis of staging.

-	-	Clinical Prognostic Stage			
-	-	0	I	II	III + IV
**Pathological prognostic stage**	**0**		1.000	<0.001 *	<0.001 *
	**I**	0.459		<0.001 *	<0.001 *
	**II**	<0.001 *	<0.001 *		1.000
	**III + IV**	<0.001 *	0.004 *	1.000	

*p*-Values of Dunn-Bonferroni test between two corresponding groups are shown in the cross table. The upper rows show the results of the clinical prognostic stage (blue letters), and the lower rows show the results of the pathological prognostic stage. For instance, *p* = 1.000 for stage 0 vs. I in the clinical prognostic stage and *p* = 0.459 for stage 0 vs. I in the pathological prognostic stage. * Statistically significant.
